# Adult-onset idiopathic opsoclonus-myoclonus syndrome

**DOI:** 10.5935/0004-2749.2022-0024

**Published:** 2023-03-08

**Authors:** Xiaohan Zhang, Wenjing Yan, Yuqiang Song, Haifang Zhu, Yanping Sun

**Affiliations:** 1 Department of Neurology, The Affiliated Hospital of Qingdao University, Qingdao, Shandong, China

**Keywords:** Opsoclonus-myoclonus syndrome/diagnosis, Opsoclonus-myoclonus syndrome/drug therapy, Immunotherapy/methods, Human, Síndrome de opsoclonia-mioclonia/diagnóstico, Síndrome de opsoclonia-mioclonia/tratamento farmacológico, Imunoterapia/métodos, Humanos

## Abstract

**Purpose:**

Opsoclonus-myoclonus syndrome is extremely uncommon in adults with an autoimmune pathophysiology. Because of the rarity of the syndrome, international recognition of opsoclonus-myoclonus-ataxia syndrome needs to be improved urgently. Therefore, the goal of this study was to raise the awareness of the opsoclonus-myoclonus-ataxia syndrome and help doctors in better diagnosing and using immunotherapy.

**Methods:**

We present a case study of an adult-onset case of idiopathic opsoclonus-myoclonus syndrome characterized by spontaneous arrhythmic multidirectional conjugate eye movements, myoclonus, ataxia, sleep disorders, and intense fear. Additionally, we conduct a literature search and summarize the pathophysiology, clinical presentation, diagnosis, and treatment of opsoclonus-myoclonus-ataxia syndrome.

**Results:**

Immunotherapies successfully treated the patient’s opsoclonus, myoclonus, and ataxia. Further, the article also includes an update summary of the opsoclonus-myoclonus-ataxia syndrome.

**Conclusion:**

The prevalence of residual sequela in adults with opsoclonus-myoclonus-ataxia syndrome is low. Early diagnosis and treatment may result in a better prognosis. Furthermore, combined immunotherapy is expected to reduce the incidence of refractory and reoccurring opsoclonus-myoclonus-ataxia syndrome.

## Abstract (pt)

**Objetivo:**

A síndrome de opsoclonia-mioclonia é extremamente rara em adultos e tem uma fisiopatologia autoimune. Devido à raridade dessa síndrome, o reconhecimento da síndrome de opsoclonia-mioclonia-ataxia precisa melhorar urgentemente em todo o mundo. Assim sendo, este estudo visou aumentar a conscientização sobre a síndrome de opsoclonia-mioclonia-ataxia e ajudar os médicos para um melhor diagnóstico e o uso correto da imunoterapia.

**Métodos:**

Este é o relato de um caso adulto de síndrome de opsoclonia-mioclonia idiopática com movimentos oculares conjugados, multidirecionais, arrítmicos e espontâneos, mioclonia, ataxia, distúrbios do sono e medo intenso. Além disso, foram pesquisadas as publicações recentes relevantes e resumiu-se a fisiopatologia, a apresentação clínica, o diagnóstico e o tratamento da síndrome de opsoclonia-mioclonia-ataxia.

**Resultados:**

A paciente recuperou-se totalmente da opsoclonia, da mioclonia e da ataxia através de imunoterapia. O artigo também fornece um resumo atualizado sobre a síndrome de opsoclonia-mioclonia-ataxia.

**Conclusão:**

Adultos com síndrome de opsoclonia-mioclonia-ataxia têm uma baixa frequência de sequelas residuais. O diagnóstico e o tratamento precoces podem levar a melhores prognósticos. Espera-se que a imunoterapia combinada reduza a incidência da síndrome de opsoclonia-mioclonia-ataxia refratária e recorrente.

## INTRODUCTION

Opsoclonus-myoclonus-ataxia syndrome (OMS) is an acute or subacute disease of the central nervous system (CNS) that primarily affects toddlers aged 12-18 months^(1)^. It is distinguished by chaotic and rapid eye oscillations, multifocal muscle jerks and severe ataxia. The incidence of OMS in children is 0.2 per 1 million per year, with adults having lower incidence rate than children^(2)^. OMS causes are classified as paraneoplastic or idiopathic, with the former accounting for half of all cases. Adults and children have different tumors associated with OMS. When compared to central nervous system tumors in toddlers, particularly neuroblastoma (NB), OMS is most commonly associated with small-cell lung cancer and breast cancer in adults^(3)^. Idiopathic causes are more common in adults and are usually caused by infection. Mycoplasma pneumonia, salmonella enterica, HIV, hepatitis C virus, rotavirus, chickenpox, and mumps virus are among the most common infectious causative agents reported in the literature^(4^-^7)^. Furthermore, the most recent research indicates that OMS is linked to coronavirus disease 2019 (COVID-19)^(8^,^9)^. Although the exact cause of OMS is unknown, we believe it is an immune dysregulation disorder. Immunosuppressive agents, such as corticosteroids, corticotropin-releasing hormone, immunoglobulin, or rituximab, are currently used to improve the quality of life in patients with idiopathic OMS.

## CASE REPORT

A 33-year-old woman came to our clinic after experiencing vertigo and nausea for a week. These symptoms quickly progressed to unsteadiness of gait and dysphagia. After the first week in the hospital, she began to have sleep problems. Spontaneous nystagmus (Video 1), broad-based gait, tendon hyperreflexia, instability in the finger-to-nose test, and a positive Romberg’s sign were among the positive neurological manifestations. The patient had a history of an upper respiratory infection 15 days prior to admission, with a sore throat and paroxysmal headache. The results of the laboratory tests revealed that tumor markers, infectious indices, and rheumatism and immunity markers were all normal. CSF analysis revealed 20 WBCs/mm^3^ of WBCs, normal glucose levels, and mildly elevated total protein (638 mg/dL, normal range 120-600 mg/L). With elevated IgG >111 mg/L (normal, 34), oligoclonal bands (OCBs) were found in CSF but not in blood. IgM greater than 4.63 mg/L (normal, 1.3) and IgA greater than 9.99 mg/L (normal, 34). Microbiological tests, which included tests for bacteria, cryptococcus, and spirochetes, came back negative. HSV (herpes simplex virus) serological testing revealed the presence of both IgM and IgG antibodies. Non-enhanced and enhanced brain MRIs, as well as breast, abdominal, and pelvic computer tomography (CT), revealed no malignancies. We considered lesions in the brainstem and cerebellum based on the manifestations and laboratory test results and made a presumptive diagnosis of rhombencephalitis. According to the suspected etiologies, the patient was immediately given empiric therapy with steroids (methylprednisolone 80 mg/day) and antiviral medicine (ganciclovir 30 mg/day). Clonazepam was prescribed to help her sleep problems. Following one week of treatment, the patient’s neurological symptoms of ataxia and opsoclonus significantly improved. After another week on prednisolone 40 mg/d, the patient was discharged home.

**Video 1** Spontaneous nystagmus.

The patient returned to our clinic two weeks later with severe psychiatric disturbance. Minor events caused her to experience intense fear. At the one-month follow-up, residual deficits (ataxia and opsoclonus) had improved. We continued prednisolone 60 mg/day for two weeks before gradually reducing the dosage. After one year of convalescence, the patient had no evidence of underlying malignancies and had almost fully recovered from opsoclonus, myoclonus, and ataxia.

### Presentation and diagnosis

As a subtype of OMS, paraneoplastic OMS (P-OMS) and idiopathic OMS have nearly identical clinical presentations (I-OMS). The majority of patients present with nonspecific nausea, vomiting, fatigue, dizziness, vision abnormalities, tremulousness, balance difficulties, and language impairment at first^(10)^. From the onset of the disease to the appearance of neurological symptoms, particularly abnormal eye movement, the latent period is approximately one month.

Opsoclonus is defined as arrhythmic, involuntary, multidirectional conjugate eye movements that do not have intersaccadic intervals^(11^,^12)^. These movements occur at a high frequency of 10-25 Hz and have an amplitude of 5-10 degrees. Opsoclonus worsens with fixation or random movement and continues during sleep or eyelid closure^(13)^. Patients frequently complain of oscillopsia, blurred vision, and vertigo as a result of this condition. It is worth noting that opsoclonus is frequently confused with ocular flutter and nystagmus. Opsoclonus and ocular flutter are both unwanted saccades that disrupt steady fixation. Opsoclonus differs from ocular flutter in that the saccade consists of several planes, including horizontal, vertical, and torsional components, rather than just the horizontal plane^(14^,^15)^. Opsoclonus is a saccade, as opposed to nystagmus, which is a rhythmic and slow oscillation that takes the eyes away from the target^(16^,^17)^. Opsoclonus can occur on its own, but it is frequently associated with other clinical manifestations such as myoclonus and ataxia.

Myoclonus manifests as sudden, shock-like, multifocal spasms that worsen in the standing position and improve in the lying position^(18)^. During sleep, small-amplitude jerks can occur^(19)^. These patterns are more common in the extremities than in the axial region (craniocervical region or trunk)^(20^,^21)^. Unsteadiness and dysphagia are common symptoms of myoclonus, which has a variety of clinical manifestations.

OMS also includes non-motor symptoms like irritability and sleep disturbances. During clinical follow-up, children with OMS typically exhibit irritability and developmental regression^(22)^. Adults with OMS, on the other hand, frequently experience sleep disturbances, including severe insomnia and frequent awakenings^(1)^.

Opsoclonus, myoclonus/ataxia, behavioral change and/or sleep disturbance, and neuroblastoma are the diagnostic criteria. OMS can be diagnosed when three of four symptoms occur. Despite the existence of diagnostic criteria, delayed diagnoses and misdiagnoses are common. This is due to atypical presentations, clinicians unfamiliar with the condition, intermittent or late opsoclonus, and the failure to recognize behavioral changes and sleep disturbances^(23)^. The time between diagnosis and treatment is approximately 11 weeks^(24)^. Atypical presentation, in the absence of opsoclonus, is nearly identical to acute cerebellar ataxia. OMS should be considered over acute cerebellar ataxia in the following circumstances: first, ataxia is not significantly improved or fluctuates over time; second, sleep disturbances appear during the course of illness; and third, ataxia is not significantly improved or fluctuates over time^(25)^.

The first step for all patients with OMS is a thorough diagnostic evaluation for the underlying tumor. There are three reasons for this: first, a high incidence of underlying tumors; second, partial symptom relief through tumor excision; and finally, different treatment principles between tumor and infection. To rule out underlying tumors, all patients should have CT scans of the chest, abdomen, and pelvis, as well as brain magnetic resonance imaging (MRI) and positron emission tomography (PET). If the imaging is negative, the work-up should be repeated every three months because certain neoplasms can manifest later than the clinic symptom. Brain MRI is usually normal in the early stages, but recent reports suggest that cerebellar atrophy is common in follow-up^(22)^. 18F-FDG positron emission tomography (18F-FDG PET) has a high diagnostic value for ruling out neuroblastic tumors^(26)^.

In most OMS patients, the CSF has a normal cell count and a mean CSF protein concentration of 52 mg/dL (normal range 15-45 mg/dL)^(11)^. Inflammatory markers in the CSF, such as the IgG index or oligoclonal bands, are present in up to 58% of patients, indicating a good prognosis with treatment^(27^,^28)^. The antibodies in the CSF lack specificity and are more commonly associated with tumors than with OMS.

The electromyogram (EMG) reveals synchronous discharges lasting less than 100ms, correlating with clinical myoclonus, and somatosensory evoked potential amplitudes and long-latency responses are normal^(29)^. Opsoclonus was found to be independent of myoclonus using periorbital bipolar electrodes.

### Immunopathogenesis

According to popular belief, OMS is an autoimmune disease mediated by humoral and cellular immunity^(30)^.

Humoral immune: Autoantibodies in paraneoplastic OMS patients were the first indication of immune involvement in OMS. Anti-Ri, anti-Hu, anti-Ma1, anti-Ma2, anti-CRMP5, anti-GlyR, anti-NMDAR, anti-GABA2R, and anti-DPPX antibodies are all known. Anti-LGI-1^(31)^ and anti-Kelch-like protein-11 antibodies^(32)^. The autoimmune response in paraneoplastic OMS is commonly thought to be caused by a cross-reactivity between neuronal tissue and certain antibodies induced by cancer, as both tissue and cancer contain the same antigens. In addition to paraneoplastic antibodies that cause autoimmune disease, the discovery of Anti-N-methyl-D-aspartate (NMDA) receptor seroconversion following herpes simplex encephalitis lends credence to the link between parainfectious antibodies and OMS^(33^,^34)^. A case of OMS in a postpartum period recently revealed Myelin oligodendrocyte glycoprotein-immunoglobulin G (MOG-IgG) positivity, implying that fetal microchimerism exposure can also initiate the immune response^(35)^. Many antibodies have been found in the serum of OMS patients, but they are not specific to OMS. For example, the glutamate receptor delta 2 antibody was found in the serum of OMS patients and was thought to be a potential biomarker of the disease. However, as the study progressed, the GluD2 antibody was not found in most OMS patients and was not specific, indicating that syndrome-specific antibodies must be discovered further. As a result, comprehensive serological tests for detecting OMS-specific antibodies are required and would aid in clinic diagnosis^(36)^. In addition to antibodies against normal nervous tissues in OMS, the presence of oligoclonal bands in CSF indicates involvement in immune dysfunctions. OMS in Aicardi-Goutières syndrome is associated with increased type 1 interferon signaling, implying that immune dysregulation is involved in the pathogenesis of OMS^(37)^.

Cellular immunity: T cells have been overlooked for years, owing to rare neuronal degeneration or scattered T cell infiltrates discovered in autopsies. Evidence of T cell population expansion in the CSF of children with OMS has changed people’s minds^(38)^. Rapid increases in CD4+ cell counts in HIV-infected patients support the role of T cells in OMS^(39)^. Although elevated neopterin in CSF does not have the sensitivity to be a biomarker, it can support T-cell activation and cell mediated immunity in OMS^(40)^. The success of B-cell-based immunotherapies and the discovery of cerebrospinal fluid lymphocytic pleocytosis support the contribution of immune cells to OMS immunopathogenesis^(41)^.

### Pathophysiology

Opsoclonus’ mechanism is unknown. The brainstem theory and the cerebellar theory are the two hypotheses. Normal saccades necessitate the involvement of the saccadic system, which is located in the brainstem. The system employs both burst neurons and omnipause neurons. The activity of burst neurons projects onto ocular motoneurons, causing saccades to be generated. It also receives projections from the fastigial nuclei of the cerebellum (FN). Omnipause neurons prevent unwanted saccades from occurring in any direction. Omnipause neurons receive neuronal projections from the mesencephalic reticular formation, the superior colliculus, and other areas. Saccadic oscillations are indicated by abnormal eye movements. Diseases affecting the saccadic system and superior regulatory center could theoretically cause saccadic oscillations^(42)^.

According to the brainstem theory, opsoclonus can be caused by increased burst neuron excitability and decreased omnipause neuron inhibition^(43)^. According to Chaumont et al., COVID-19-associated abnormal eye movement is caused by brainstem dysfunction^(44)^. Omnipause neurons use glycine as a neurotransmitter, and anti-GlyR antibodies can change the inhibitory properties of saccadic burst neurons. The discovered antibodies bolster the case for brainstem dysfunction. The cerebellar possibility is that saccadic oscillation is caused by FN disinhibition. Functional MRI evidence demonstrated bilateral activation of the fastigial nuclei^(45)^. Autopsies have long revealed a loss of Purkinje cells or demyelination in the cerebellum. An electrophysiology study found that opsoclonus-myoclonus originates in the brainstem, with concurrent abnormalities in cerebellar circuits^(29)^. These various theories are clearly not independent, but rather have influential relationships.

### Treatment

Patients should begin with tumor resection because it can help alleviate some of the symptoms. In the experience of treating OMS secondary to NB, research has shown that NB resection does not resolve all of the symptoms of OMS, but combined immunotherapy has a satisfactory outcome. As a result, they advocated for aggressive immunotherapy treatment of tumor patients^(46)^.

Given the likely immune dysfunction that underpins OMS, immunosuppression plays an important role in affected individuals^(47)^. As hypothesized immunopathogenesis, the immune activation inducing by immune cells, the therapy will proceed as follows: first, limiting T/B cell production and accelerating T/B cell removal. Second, prevent immune activation. The most common medications are adrenocorticotropic hormone (ACTH), corticosteroids, and intravenous immunoglobulins (IVIG).

Because standard drugs are less effective against neuropsychological disturbances and there is an increased subset of CSF lymphocytes in patients who fail primary treatment, alternative treatments based on an elevated B/T count, such as rituximab, cyclophosphamide, and methotrexate, are gaining traction. Rituximab, an anti-CD20 monoclonal antibody that inhibits B cell expansion, is generally approved for second-line treatment of refractory and reoccurring OMS as a broad-acting immunosuppressive agent^(48)^. This medication has the advantage of shortening the duration of steroid use and improving prognosis. However, rituximab has been shown to increase the risk of infection and tumor growth^(49)^. Lower rituximab doses may not reduce total risk, so patients with normal B cell counts may not require rituximab. However, according to one study, low doses of rituximab (300 mg/m^2^) in rituximab combination immunotherapy are just as effective as the standard dose^(50)^. Rituximab improves prognosis by lowering the risk of disability^(51)^.

Despite the fact that abnormal T cells are the underlying pathomechanism of OMS, the treatment of targeting T cells is ineffective. Some researchers demonstrated that mycophenolate mofetil can reduce T cell CSF expansion (-40%) in OMS but has no effect on relapse prevention^(51)^. Cyclophosphamide and methotrexate, cytostatic agents that affect both T and B cells, are also effective in the treatment of OMS. However, due to a lack of clinical statistics, the standard of care has not been established, making the selection of appropriate drugs difficult.

Clonazepam (3 mg/day), baclofen (45 mg/day), and levetiracetam (3000 mg/day) are common symptomatic treatments used in conjunction with immunotherapy. Clonazepam is prescribed for patients who have significant opsoclonus and severe sleep disturbances^(52^,^53)^. In the treatment of myoclonus, zonisamide (300 mg/d to 500 mg/d divided into 1 or 2 daily doses) and anticholinergics can be tried^(54)^.

### Prognosis

Delayed diagnosis and chronic relapse can result in long-term motor and cognitive consequences. The length of time between symptom onset and diagnosis (more than 2 months) is associated with late-occurring neurological and neuropsychological long-term deficits^(22)^. Brunklaus et al. retrospectively evaluated 101 patients with OMS and concluded that severe initial symptoms, lack of remission at the last follow-up, and chronic relapses were associated with a poor long-term cognitive prognosis^(55)^. Pediatric patients with OMS are more likely to have residual neurological sequelae: such as cognitive impairment, behavioral abnormalities, and language disorders^(55^,^56)^. Adult sequelae, in contrast to childhood sequelae, are relatively benign and include gait ataxia and residual dysarthria^(57)^. Chronic relapse is frequently associated with a decreased effect on medical therapy, and there is a progressive worsening of neurological symptoms per OMS relapse^(58)^.

Moreover, achieving clinical remission and reducing recurrence rates can greatly improve the patients’ quality of life^(24)^. Relapsing can be triggered by a simple infection or dosage reduction. Pranzatelli et al. analyzed 159 children with OMS recurrences, finding that 40% were infection-related, 48% by gradual drug reductions, and 12% by both infection and treatment^(50)^.

Adult sequelae, in contrast to childhood sequelae, are relatively benign and include gait ataxia and residual dysarthria^(57)^.

The prognosis of paraneoplastic and idiopathic OMS differed. The studies suggested that idiopathic OMS appears to be self-limiting. More than half of patients with paraneo-plastic OMS have more relapses and a worse outcome. Approximately 70% of patients have ongoing neurological, mental, cognitive, and behavioral sequelae^(57)^.

## DISCUSSION

OMS is uncommon in populations and even more so in adults. It is distinguished by opsoclonus, myoclonus, and ataxia^(57)^. Patients with adult-onset OMS are more likely to develop small-cell lung cancer and breast cancer, and young adult women are more likely to develop occult ovarian neoplasms^(58^,^59)^. When paraneoplastic serological and imaging evaluations are negative, infection as the second leading cause should be considered. Positive serum antibodies to herpes simplex virus (HSV) immunoglobulin G (IgG) in this case indicated HSV infection within the last three months. Although serum antibody testing is used to diagnose HSV infection, polymerase chain reaction (PCR) detection is the only mature technology for detecting virus activity. Unfortunately, we did not perform CSF PCR in our case.

HSV is a common pathogen that infects a large percentage of the human population. HSV infections are typically recessive and localized, despite the fact that the virus appears to be the primary cause of scattered viral encephalitis syndrome. Through latency in neurons, HSV can maintain a lifelong infection^(17)^. When the host’s immunity declines or is stimulated by stress factors, the virus replicates to form a recurring infection that causes encephalitis^(18)^. It has been demonstrated that interactions between host immunity and CNS cells following HSV exposure can result in OMS. The literature on OMS caused directly by HSV infection is limited. If untreated, HSV encephalitis has a high mortality rate. Immunotherapies combined with antimicrobial therapy can reduce HSV-related mortality in OMS. Therefore, we recommend empiric antiviral therapy in cases where there is a high suspicion of viral infection. Based on the PCR results, the antiviral drug can be changed.

The presence of oligoclonal bands in both positive CSF and negative serum samples suggested intrathecal invasion of central nervous system tissues, as seen in autoimmune encephalitis. The routine laboratory CSF testing results show mild and nonspecific increase in protein levels. Both are indicative of inflammatory or immune-mediated pathogenesis. Although the cause is unclear, no tumor was found in this young woman after 1 year of follow-up, allowing us to investigate whether OMS is caused by postinfectious or parainfectious in our case.

OMS is related to rhombencephalitis and may be one of the symptoms of the latter. Rhombencephalitis is an umbrella term for inflammatory diseases of the pons, cerebellum, and medulla oblongata^(60)^. Rhombencephalitis symptoms can be similar to those of OMS. OMS is characterized by opsoclonus, myoclonus, and ataxia all at the same time.

We stopped using antiviral drugs and tried to reduce the amount of steroid to avoid side effects after the rapid administration of methylprednisolone and ganciclovir relieved neurological symptoms. It is unclear whether immunosuppressive therapy alone or in combination with an antiviral drug resulted in clinical remission.

During the tapering, she visited our clinic again for specific psychological and behavioral abnormalities. The literature focuses on movement disorders in recurrence but not on the presence of psychiatric symptoms. We assumed that such a condition was the result of late events or a relapse caused by insufficient drug load. We also suspected that the aforementioned symptoms were immune-mediated, so, we tried methylprednisolone 80 mg/day and had excellent results in treating the psychiatric symptoms.

In conclusion, we reported a parainfectious OMS. This case raises awareness of OMS and helps doctors in making better diagnoses and using immunotherapy correctly.
